# A single course of lusutrombopag for multiple invasive procedures in cirrhosis-associated thrombocytopenia: A case series

**DOI:** 10.1097/MD.0000000000031429

**Published:** 2022-11-04

**Authors:** Marco Biolato, Federica Vitale, Giuseppe Marrone, Luca Miele, Antonio Grieco

**Affiliations:** a Fondazione Policlinico Universitario Agostino Gemelli IRCCS, Roma, Italy; b Catholic University of Sacred Heart, Rome, Italy.

**Keywords:** case report, liver disease, platelet, procedures, TPO-agonists

## Abstract

Lusutrombopag is a thrombopoietin receptor agonist which reduces the need for platelet transfusions before planned invasive procedures. A post hoc analysis of data from the registration trials observed that lusutrombopag-treated patients who achieved a platelet count > 50 × 10^9^/L (responder patients) did so in a median of 6 days and the effect on platelet count lasted for nearly 3 weeks in total. Since patients with cirrhosis often require repeat invasive procedures, this kind of response-time trend sheds light on the possibility of placing more than one invasive procedure within a single course of lusutrombopag treatment.

**Patient concerns:**

Platelet transfusion represents the gold standard in this setting, but is limited by the risk of adverse events and limited availability.

**Diagnoses:**

We describe our experience with lusutrombopag in three patients with severe cirrhosis-associated thrombocytopenia who underwent multiple invasive procedures after a single course of treatment.

**Interventions:**

The treatment schedule is lusutrombopag orally 3 mg/daily for 7 days and then a time window of 6 days (day 9–14) for the elective invasive procedure.

**Outcomes:**

All three patients achieved good response to lusutrombopag treatment and were able to undergone more invasive procedures in the same course of treatment without need of platelet transfusion.

**Lessons:**

our preliminary experience supports the safety and the effectiveness of lusutrombopag in patients with severe cirrhosis-associated thrombocytopenia who underwent multiple invasive elective procedures after a single course.

## 1. Introduction

Severe thrombocytopenia (platelet count < 50 × 10^9^/L) can occur in patients with cirrhosis and has been associated with increased bleeding risk and affects the feasibility and prognosis of invasive procedures.^[1]^ Still, nowadays, platelet transfusion represents the gold standard to raise the platelet count before invasive scheduled procedures in this patient population.^[2]^ Nevertheless, platelet transfusion, far from being completely safe, also involves the risk of adverse events such as transfusion reactions, infections, volume overload, transfusion-related acute lung injury, and refractoriness.^[3]^

Nowadays, second-generation thrombopoietin receptor agonists represent a therapeutic option for the treatment of cirrhosis-associated thrombocytopenia.^[4]^ Lusutrombopag is an orally bioavailable molecule that acts selectively on the human thrombopoietin receptor and activates signal transduction pathways promoting the proliferation and differentiation of bone marrow cells into megakaryocytes.^[5]^ In two phase 3, randomized, double-blind studies (Lusutrombopag for Thrombocytopenia in Patients With Chronic Liver Disease Undergoing Elective Invasive Procedures 1 [L-PLUS 1] and Lusutrombopag for Thrombocytopenia in Patients With Chronic Liver Disease Undergoing Elective Invasive Procedures 2 [L-PLUS 2]), lusutrombopag was superior to placebo for reducing the need for platelet transfusions and achieved durable platelet count response in patients with cirrhosis-associated severe thrombocytopenia undergoing invasive procedures, with a safety profile similar to placebo.^[6,7]^ Therefore the approved treatment schedule is lusutrombopag orally 3 mg/daily for 7 days and then a time window of 6 days (day 9–14) for the elective invasive procedure.

Few data are available about the efficacy and safety of multiple invasive procedures performed in a single treatment course with lusutrombopag. In this case series, we describe our experience with lusutrombopag in three patients who underwent multiple invasive procedures after a single course of treatment.

## 2. Description

### 2.1. Patient 1

**Figure 1.**
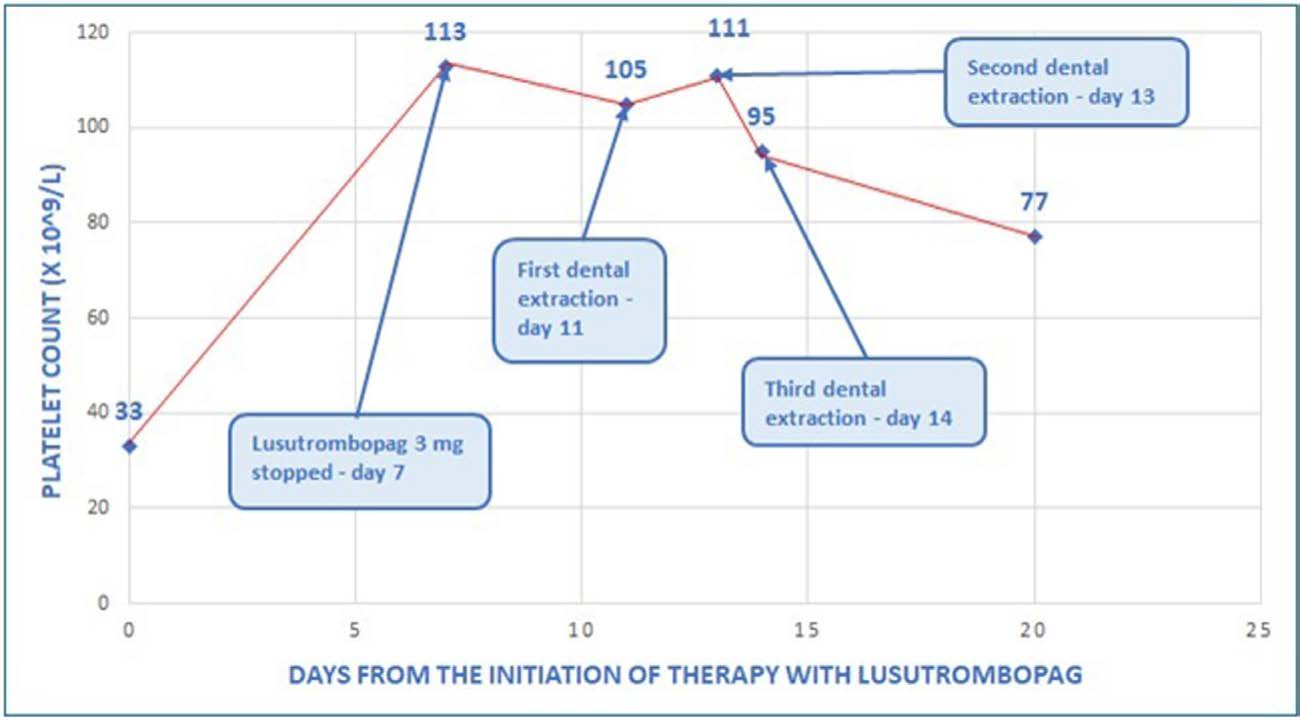
Platelet count trend in patient 1.

A 62-year-old woman with a history of autoimmune liver cirrhosis, Child-B9 stage, model for end-stage liver disease (MELD) score of 22, in February 2022 underwent diagnostic work-up before waiting list registration for liver transplantation. She performed a panoramic dental X-ray and the dentist advised her that dental extractions were necessary to prevent potential infections. Her platelet count was 33 × 10^9^/L and her spleen diameter was 15.8 cm. The portal vein system was patent. She started lusutrombopag 3 mg daily for 7 days and her platelet count rose with a peak of 113 × 10^9^/L on day 7. The first dental extraction session was done on day 11 with a platelet count of 105 × 10^9^/L, the second session on day 13 with a platelet count of 111 × 10^9^/L, and the third session on day 14 when her platelet count was still 95 × 10^9^/L (Fig. 1). No platelet transfusions were required before or after the procedures and no bleeding or thrombotic complications occurred. Moreover, platelets were still 71 × 10^9^/L after 73 days from the beginning of treatment. A computed tomography scan performed three months after lusutrombopag treatment showed no portal vein thrombosis. The patient was registered on the waiting list for transplantation, but died before transplant.

### 2.2. Patient 2

**Figure 2.**
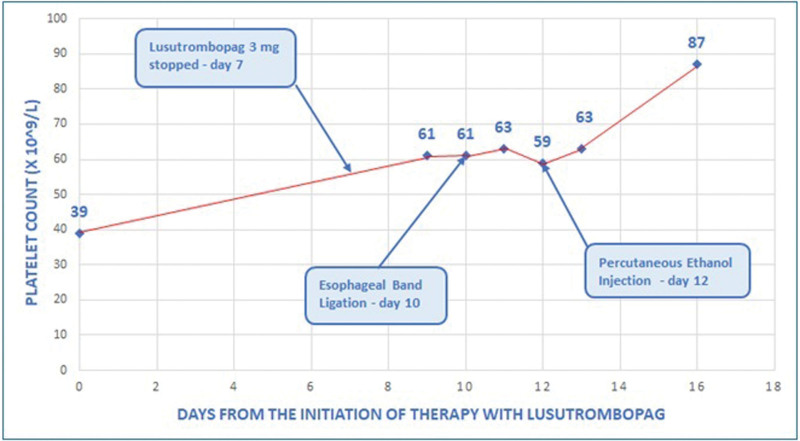
Platelet count trend in patient 2.

A 51-year-old man with a history of diabetes mellitus, moderate obesity (body mass index 37), and metabolic cirrhosis, Child-A6 stage, MELD score 12, was admitted to our clinic in March 2022 because of esophageal varices with a high risk of bleeding and early-stage hepatocellular carcinoma. He was a candidate for esophageal band ligation and percutaneous ethanol injection on a liver nodule of 1.8 cm in the VI segment. His platelet count was 39 × 10^9^/L and his spleen diameter was 21.9 cm. The portal vein system was patent. He started lusutrombopag 3 mg daily for 7 days. He underwent esophageal band ligation on day 10, when his platelet count was 61 × 10^9^/L, and percutaneous ethanol injection on day 12 with a platelet count of 59 × 10^9^/L (Fig. 2). No platelet transfusions were required before or after the procedures and no bleeding complications occurred. The platelet count peak was 87 × 10^9^/L on day 16. A computed tomography scan performed two months after lusutrombopag treatment showed new onset of partial portal vein thrombosis of the right branch; he was asymptomatic and started enoxaparin treatment and follow-up at our outpatient clinic.

### 2.3. Patient 3

**Figure 3.**
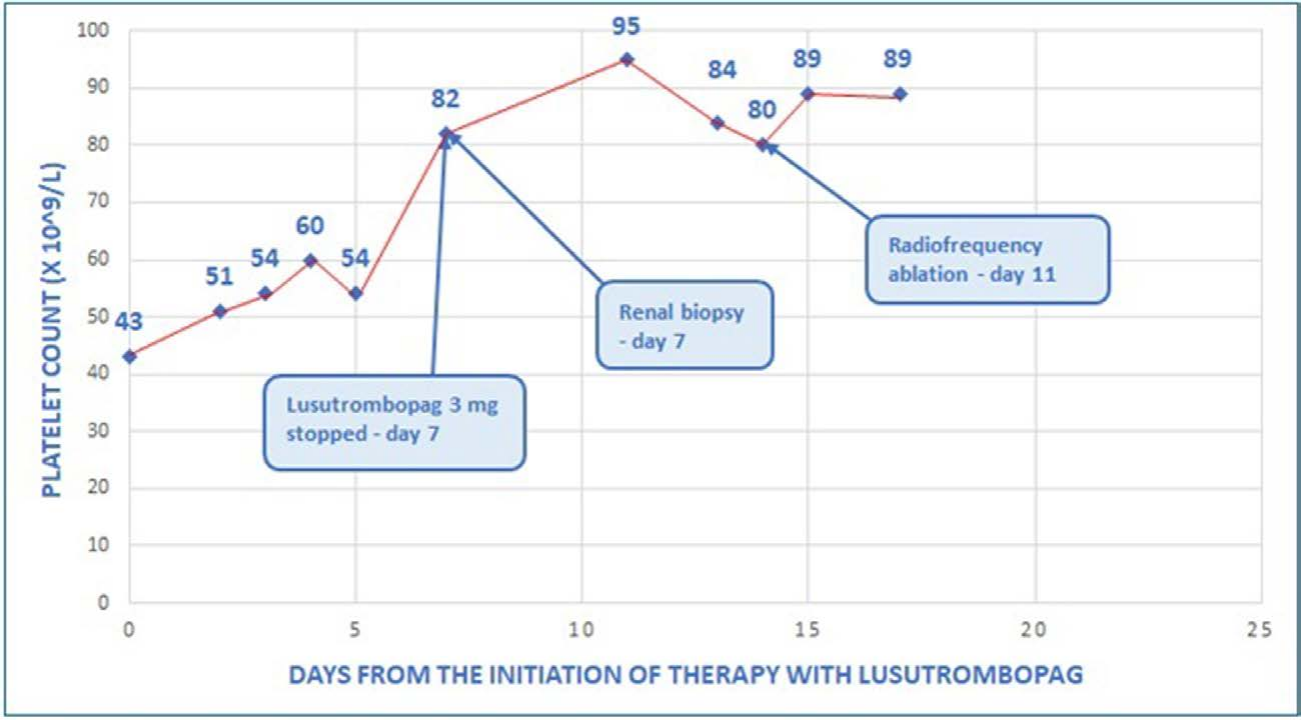
Platelet count trend in patient 3.

A 64-year-old man with a history of diabetes mellitus, alcohol use disorder, and liver cirrhosis, Child-A6 stage, MELD score of 17 complicated by early-stage hepatocellular carcinoma, was referred for liver transplantation on April 2022. He presented an acute renal failure associated with red blood cells detected in his urinalysis. He was a candidate for a kidney biopsy. His platelet count was 43 × 10^9^/L and his spleen diameter was 18.5 cm. The portal vein system was patent. He started lusutrombopag (3 mg daily for 7 days) and he underwent a kidney biopsy on day 7 with a platelet count of 82 × 10^9^/L. Then, he underwent radiofrequency ablation of a liver nodule of 2.4 cm in IV segment on day 11 when his platelet count was 80 × 10^9^/L (Fig. 3). No platelet transfusions were required before or after the procedures and no bleeding or thrombotic complications occurred. Kidney biopsy showed tubular necrosis and renal function improved with conservative management. A computed tomography scan showed a complete radiological response on the liver nodule. He goes on the evaluation process for listing.

## 3. Discussion

Undoubtedly, in our clinical experience, all patients showed a good response to lusutrombopag treatment. Platelet count at peak increased by three times the basal values in patient 1 and nearly twice in patients 2 and 3. Our observation fits well with that observed in a post hoc analysis of L-PLUS 1 and L-PLUS 2 trials data, where platelet count at least doubled from baseline in 52.6% of patients treated with lusutrombopag.^[8]^ Treatment response, defined as a platelet count > 50 × 10^9^/L the day before the invasive procedure, is variable, and many predictive factors have been investigated. A multivariate analysis identified that a subset of patients with a baseline platelet count < 30 × 10^9^/L were less likely to reach a maximum platelet count > 50 × 10^9^/L.^[9]^ Furthermore, patients with a higher splenic volume, assessed by software from computed tomography axial section, were less likely to have an increase in platelet count 20 × 10^9^/L from baseline.^[9]^ In another study, the response rate was 94% in patients with a baseline platelet count > 30 × 10^9^/L and 63% in patients with a baseline platelet count < 30 × 10^9^/L.^[10]^ In this study patients with a higher splenic volume had a lower response rate when the baseline platelet count was < 30 × 10^9^/L.^[10]^ A third study showed that responders patients had a lower splenic volume compared to non-responders and splenic volume was found to be an independent factor that predicted platelet response in a multivariate analysis.^[11]^ In addition, comorbidities seem to influence the response rate to lusutrombopag therapy. Nishida et al^[12]^ identified the presence of diabetes as a negative predictor of response to lusutrombopag, while Takeuchi et al^[13]^ noticed that renal function parameters (blood urea nitrogen, creatinine, glomerular filtration rate) were significantly and negatively associated with platelet count. Finally, the post hoc analysis of L-PLUS 1 and L-PLUS 2 trials by Tanaka et al^[14]^ revealed that baseline leukocyte count below the normal range is associated with smaller platelet count increases after lusutrombopag treatment than patients with leukocyte count within the normal range.

The last interesting point of our preliminary observation is the reflection on the duration of the effect of the treatments on the peripheral platelet count. According to a flow cytometry study, the half-life of transfused platelets was about 72 hours.^[15]^ In comparison, a post hoc analysis of data from the L-PLUS 1 and L-PLUS 2 trials observed that lusutrombopag-treated patients who achieved a platelet count > 50 × 10^9^/L (responder patients) did so in a median of 6 days and the effect on platelet count last for nearly 3 weeks in total.^[8]^ This kind of response-time trend sheds light on the possibility of placing more than one invasive procedure within a single course of lusutrombopag treatment. Patients with cirrhosis often require repeat invasive procedures. Sano et al^[16]^ evaluated the efficacy and safety of repeated lusutrombopag use in patients undergoing multi-cycle invasive procedures at intervals > 1 month: data about 8 patients showed how platelet counts increased significantly compared with baseline, without reduction in the efficacy of the treatment after repeated administrations. Similarly, Kawaratani et al^[17]^ demonstrated that there was no significant difference in platelet increase comparing the first and the following treatments with the medication. This evidence was confirmed by Ishikawa et al^[18]^ in a population of eight patients with hepatocellular carcinoma who had a platelet count < 50 × 10^9^/L and underwent multiple radiofrequency ablations after repeated use of Lusutrombopag. They observed that repeated use of lusutrombopag increased the platelet count, and it did not cause any serious adverse events. However, as demonstrated in our series of cases, lusutrombopag can achieve an increase in platelet count such as to allow the patient to undergo more than one elective procedure within the same cycle of treatment. No other case currently reported in the literature documents patients who underwent multiple invasive procedures within the same course of treatment.

In conclusion, our preliminary experience supports the safety and the effectiveness of lusutrombopag in patients with severe cirrhosis-associated thrombocytopenia who underwent multiple invasive elective procedures after a single course. However, other real-world studies are advisable with the aim of identifying potentially better (and prolonged) responder patients to lusutrombopag treatment.

## Author contributions

**Conceptualization:** Antonio Grieco.

**Writing – original draft:** Marco Biolato, Federica Vitale.

**Writing – review & editing:** Giuseppe Marrone, Luca Miele, Antonio Grieco.
